# Carcinoma of the Maxillary Antrum: A Case Report

**DOI:** 10.7759/cureus.2614

**Published:** 2018-05-13

**Authors:** N M Praveena, Gopla Maragathavalli

**Affiliations:** 1 Oral Medicine and Radiology, Thai Moogambigai Dental College & Hospital, Mugappair, IND; 2 Oral Medicine and Radiology, Saveetha Dental College and Hospital, Saveetha University, Chennai, IND

**Keywords:** maxillary antrum, squamous cell carcinoma, tumor

## Abstract

Maxillary sinus squamous cell carcinoma is an aggressive tumor, usually diagnosed at an advanced stage and most patients present with very poor prognosis and survival rate. We report a case of the patient who presented with pain and swelling in the left maxillary region. Due to the advanced stage at which it was presented and the involvement of vital structures, the patient was subjected to palliative treatment. The symptoms of maxillary sinus carcinoma can be non-specific, resulting in late diagnosis. It is pertinent for the maxillofacial physician to be aware of these sinus pathologies and arrive at an early diagnosis to improve the survival rate.

## Introduction

Maxillary sinus cancer is a relatively rare neoplasm with an incidence representing a small percentage (0.2%) of human malignant tumors and only 1.5% of all head and neck malignant neoplasms [1]. Asian countries report a very high incidence of maxillary sinus carcinoma, which makes it important for us to raise general awareness among oral stomatologists [2]. It predominantly occurs within the maxillary sinus (60%-70%) and less frequently in the nasal cavity (12%-25%) and the ethmoid (10%-15%) and sphenoid/frontal sinuses (1%) [3]. With respect to squamous cell carcinoma (SCC) from the maxillary sinus (MxSSCC), it affects mainly middle-aged men (55-65 years' old) from Eastern countries and has, as the major risk factors some chemicals and viruses [4-5]. Maxillary carcinoma has one of the highest incidences as compared to other paranasal sinus carcinomas, and it incurs the worst prognosis. It is important for oral physicians to understand the differential diagnosis of such lesions.

## Case presentation

**Figure 1:**
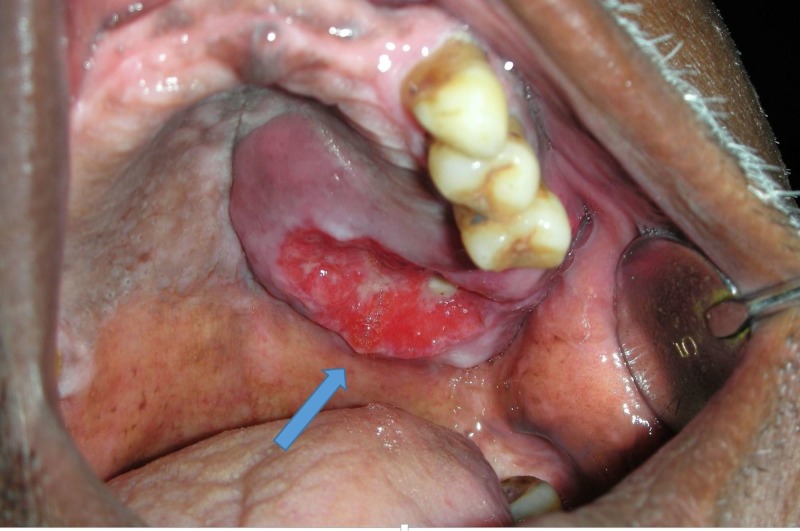
Intraoral ulceroproliferative growth involving the palate

A 55-year-old male patient reported with mild swelling and pain in the left zygomatic region. The history of present illness revealed that the patient had noticed the swelling in the last few weeks and it also had an associated intraoral ulcer. The patient was a known diabetic who was under medication. He had a habit of smoking cigarettes for the past 15 years. He smoked almost 15 cigarettes per day. The clinical examination revealed a firm swelling in the left zygomatic region measuring approximately 3 cm to 5 cm. It was mildly painful on palpation. The left maxillary region had significant paresthesia, nasal obstruction, and episodes of pain. A palpable left submandibular lymph node was present, which was also tender and fixed. The cervical lymph node on the left side was also palpable (Figure 1). The intraoral examination revealed an ulceroproliferative growth measuring 4 cm to 6 cm in dimension.

**Figure 2:**
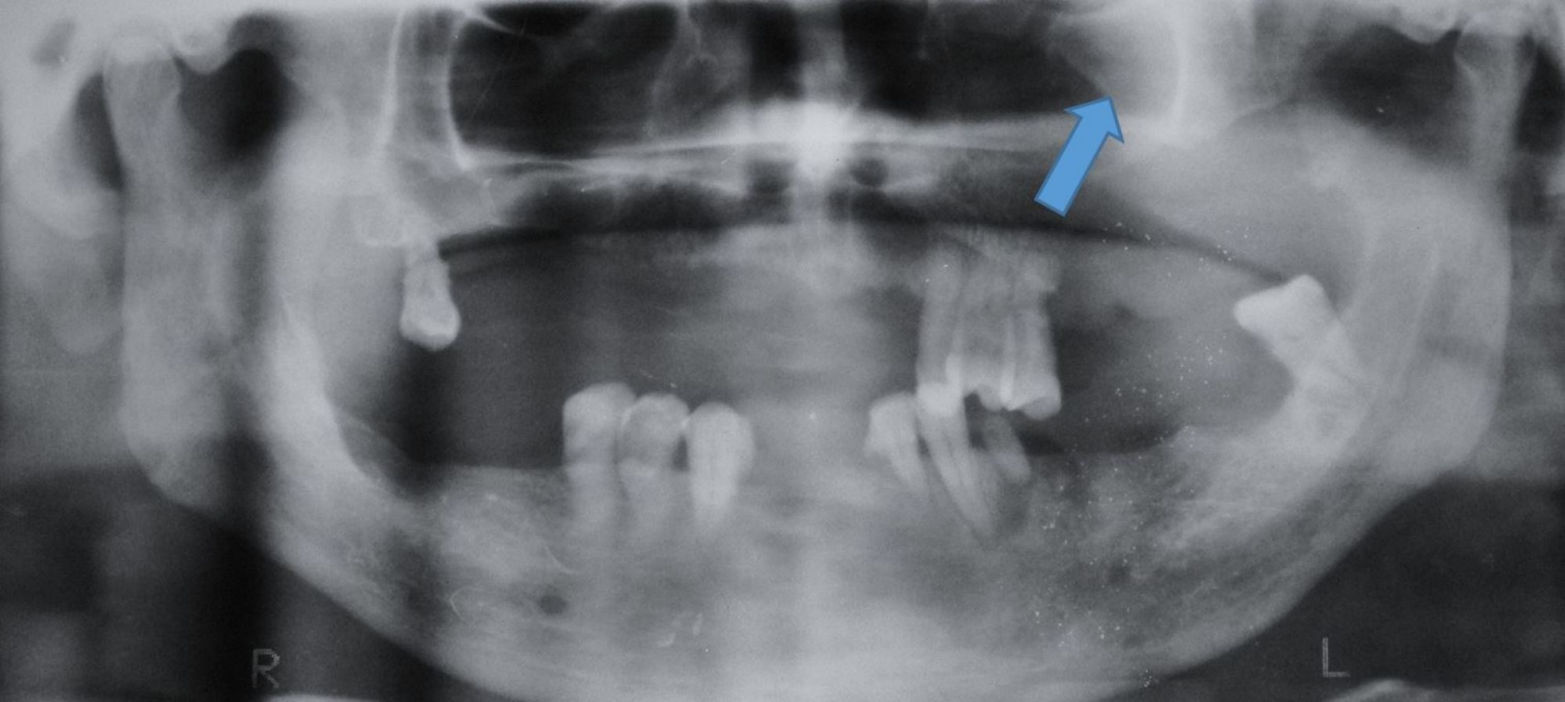
Orthopantomograph showing a soft tissue shadow in the left palate region

The lesion was extending in relation to teeth 23, 24, and 25. The ulcer was covered with necrotic slough. Purulent discharge and bleeding were present in the lesion. The other teeth in the quadrant were missing (Figure 2). A panoramic radiograph revealed extruded teeth 23, 24, 25.

**Figure 3:**
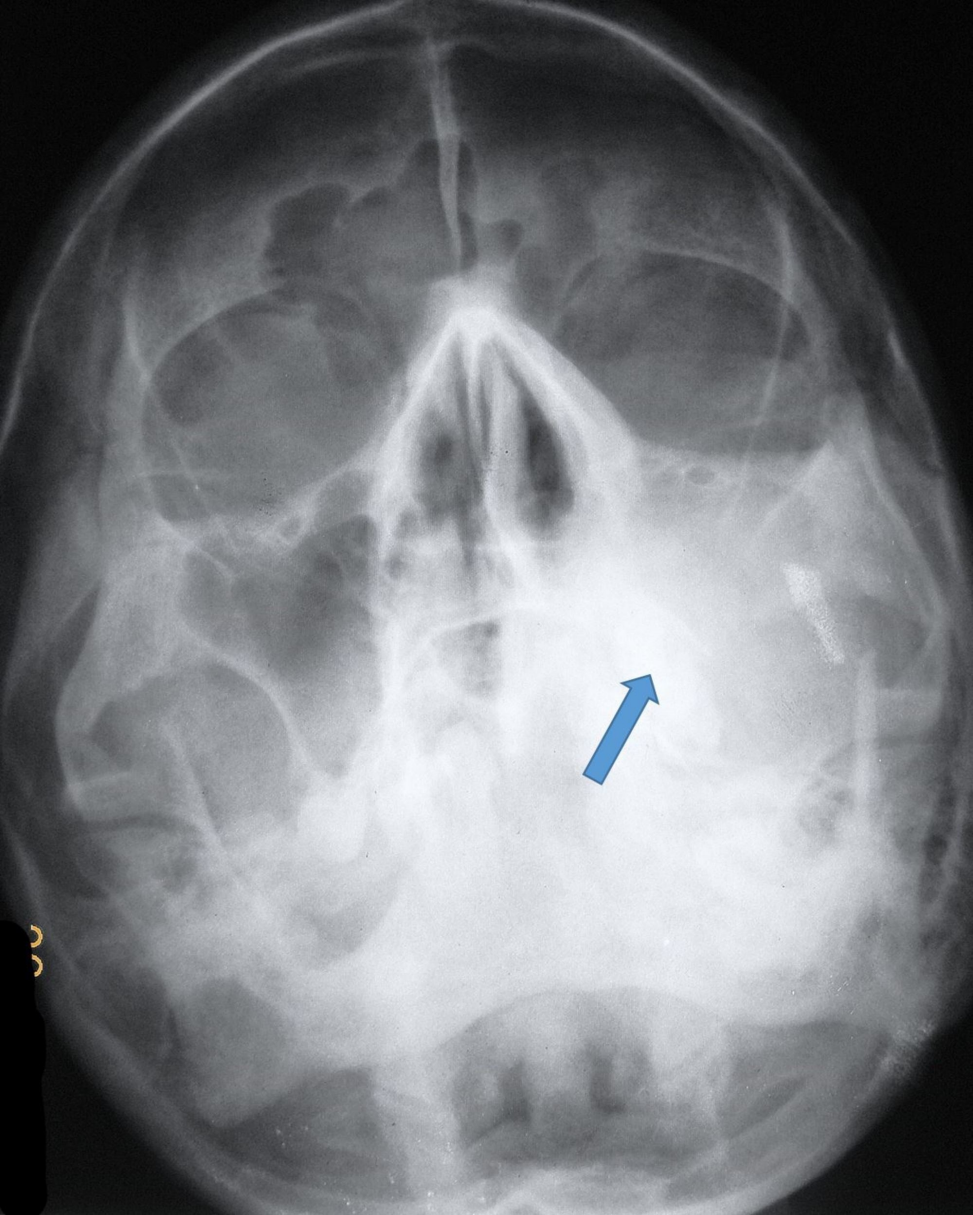
Paranasal sinus view showing the opacification of the left maxillary sinus

There was increased radiopacity in the left maxillary sinus (Figure 3). Water’s view showed the opacification of the entire left maxillary sinus.

**Figure 4:**
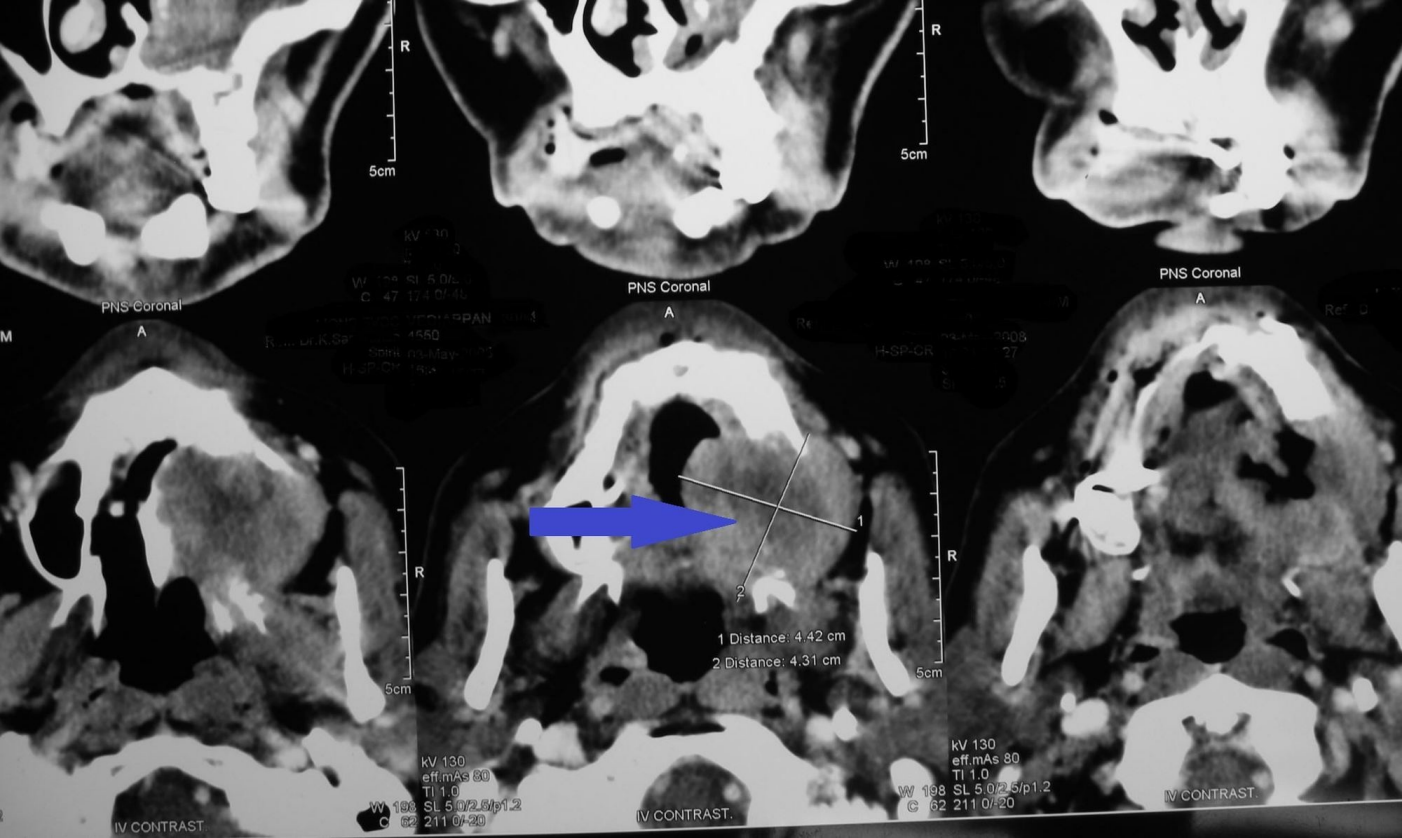
Computed tomography of the orofacial region showing heterogenous enhancement in the left maxillary sinus and destruction of the palate and left maxillary antrum

An intraoral extension of this mass was also evident. The inferior, posterior, lateral, and medial walls of the left maxillary sinus appeared to be destroyed (Figure 4). A computed tomography (CT) scan showed a lesion extending into the maxillary space and the nasal cavity. A heterodense soft tissue lesion showing heterogeneous contrast enhancement in the left maxillary sinus and hard palate with the destruction of the posterolateral wall, medial wall, and floor of the left maxillary antrum, extending into the adjacent retro maxillary space and medially extending into the left nasal cavity, obliterating all meati with the destruction of nasal turbinates. It was also inferiorly extending into the oral cavity. The CT was suggestive of carcinoma antrum. The CT was sufficient to understand the extensions of the lesions and the destruction of nasal turbinates. Further radiological investigations were, hence, not considered.

**Figure 5:**
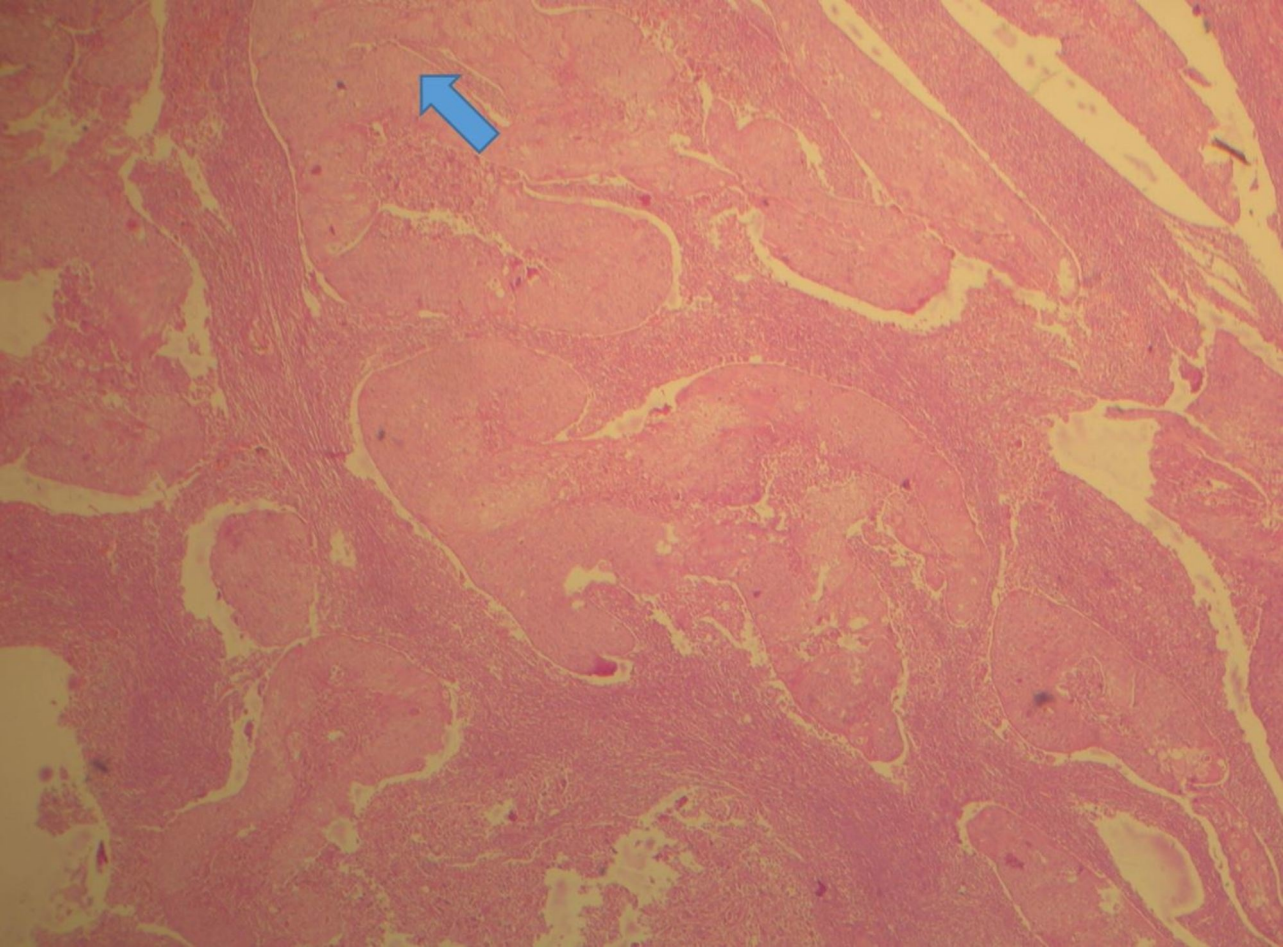
Invading epithelial islands exhibiting the dysplastic feature (H&E, 10X) H&E: hematoxylin and eosin

Considering the patient history and clinical features and the fact that patient was experiencing paresthesia, a biopsy was deemed mandatory (Figure 5). On microscopic examination, the given hematoxylin and eosin (H&E)-stained soft tissue section showed dysplastic epithelial islands arranged in sheets and nests, invading fibrovascular stroma.

**Figure 6:**
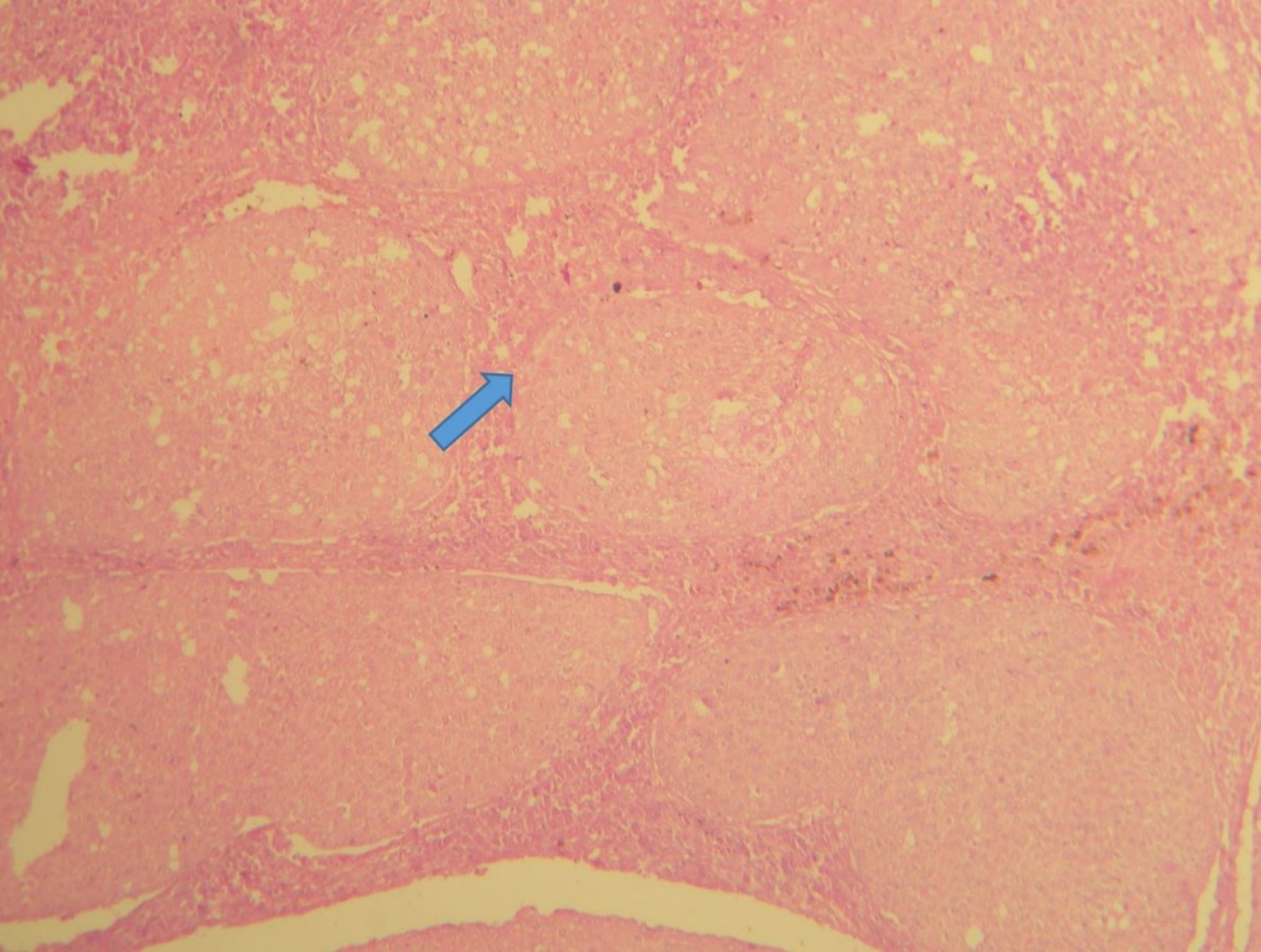
Invading epithelial islands exhibiting the dysplastic feature (H&E, 40X) H&E: hematoxylin and eosin

As shown in Figure 6, the dysplastic epithelial cells exhibited an increased nuclear-cytoplasmic ratio, individual cell keratinization, and increased mitotic figures.

Keratin pearl formation was also evident, suggestive of well-differentiated squamous cell carcinoma. The patient was referred to the maxillofacial surgery department and briefed on surgical and chemotherapeutic treatment modalities. As the patient was from a poor socio-economic background and had also reported at an advanced stage, he declined treatment and was advised palliative management. The palliative treatment mainly concentrated on the pain relief and nutritional needs of the patient. The patient was treated with acetaminophen and, later, with stronger medications like opioids to manage pain. A feeding tube was inserted into the stomach through the throat since the patient was having extreme difficulty in swallowing. Sadly, the patient succumbed within a few weeks.

## Discussion

Oral and pharyngeal malignant neoplasia is the sixth most common malignancy in the world [6]. In developing countries like India, the oral cavity and pharynx comprise the third-most-common malignancy site [7]. Carcinoma of the maxillary antrum, though, is rare. Three percent of all head and neck cancers are known to originate from the paranasal sinuses [8]. Out of these, 80% originate from the maxillary antrum and, histologically, 60%–90% of these are squamous cell carcinomas [8-9].

Oral squamous cell carcinoma is a disease with well-established risk factors, including smoking and alcohol use. Carcinoma of the maxillary sinus is very uncommon and its treatment poses several challenges to the head and neck surgeons and radiation and medical oncologists [4-5]. First, they often present in advanced stages. Second, the complex anatomy and the close proximity of critical structures make surgical excision a challenge. A majority of the patients present with advanced diseases, making prognosis poor. Our patient reported at an advanced stage and was having clear symptoms of nasal obstruction and paresthesia in the zygomatic region. Unfortunately, the patient had ignored the symptoms for quite some time. Paresthesia should be considered a pertinent sign of malignancy though it can present in certain cases of nerve damage occurring in post-surgical procedures. Hence, it is mandatory that the possibility of a malignant neoplasm be ruled out in all patients presenting with paresthesia [10].

In the present case, the ulceroproliferative growth was very much suggestive of malignancy. In a few cases, the swelling can occur like a dentoalveolar abscess and, in those cases, be easily misdiagnosed. These lesions are extremely invasive. They extend medially toward the nasal cavity; superiorly, they may invade the orbit and ethmoid sinus; anterolaterally, they may reach soft tissues; and inferiorly, they may reach the maxillary sinus floor and dental alveolus and be present as a proliferative growth in the palate. Posteriorly, they may reach the pterygopalatine fossa and pterygoid muscles. Through the pterygoid fossa, they may superiorly extend toward the orbital fissure and the cavernous sinus [11].

The most effective barrier against tumor propagation is the integrity of the periosteum that is particularly more resistant in two critical areas, which are the skull base and orbit [10]. The destruction of the maxillary sinus walls, especially the inferior antral wall, can be identified by panoramic radiography. In advanced cases, this imaging modality may not show evidence of early bone destruction. CT and magnetic resonance imaging (MRI) are the investigation of choice in such situations. The primary reason for advising CT and MRI studies in cases of maxillary antrum carcinoma is for better visualizing the invasion of structures beyond the site of origin. On CT studies, all of the cases present as soft tissue masses in the maxillary sinus cavity, with more than 70% to 90% of cases showing bony destruction [10]. CT provides more details of bone involvement than MRI. In the differential diagnosis of maxillary sinus carcinoma, it is important to include primary sinonasal neoplasms like undifferentiated carcinoma, nasopharyngeal carcinoma, lymphoma, esthesioneuroblastoma, and adenocarcinoma of minor salivary gland origin as well as metastatic diseases [10].

The risk factors associated with MxSSCC development are enormous, but chronic exposure to nickel, chlorophenol, formaldehyde, textile dust, wood, and cigarette smoking have been frequently reported [4-5]. It was observed that the risk of neck metastases significantly increased when a tumor invaded the oral cavity. Our patient had oral invasion and showed neck metastasis. The management of head and neck cancers involves exactly staging the extent of the disease (with the aid of CT or magnetic resonance imaging) in accordance with the TNM (tumor, nodes, metastases) classification of malignancy and determining if surgical resection is feasible. Early diagnosis is, therefore, paramount to a favorable prognosis. New approaches, such as neoadjuvant or concomitant chemoradiotherapy with aggressive surgery, need to be considered and evaluated in prospective cases. MxSSCC is rapidly progressing and most patients die in a span of two years' time [12-13]. The five-year survival rate has remained unaltered over many years [3].

## Conclusions

It is highly important that the oral physician understand the differential diagnosis of these swellings. Careful attention should also be paid to the symptoms of these diseases, as they can be nonspecific and ignored by the patient. An astute physician can arrive at an early diagnosis of various paranasal sinus carcinomas and immediately extend treatment. Proper investigations with CT and MRI and prompt treatment planning can greatly improve patient morbidity and mortality rates.
